# Prognostic factors for lymph node metastasis from upper gingival carcinomas

**DOI:** 10.1007/s11282-021-00568-w

**Published:** 2021-09-24

**Authors:** Mazen Aldosimani, Rinus G. Verdonschot, Yuri Iwamoto, Mitsuhiro Nakazawa, Sanjay M. Mallya, Naoya Kakimoto, Satoru Toyosawa, Sven Kreiborg, Shumei Murakami

**Affiliations:** 1 Department of Oral and Maxillofacial Radiology, Osaka University Graduate School of Dentistry, Suita, Osaka Japan; 2 Division of Radiology, Department of Oral Medicine and Diagnostic Sciences, College of Dentistry, King Saud University, Riyadh, Kingdom of Saudi Arabia; 3 Department of Oral and Maxillofacial Radiology, Institute of Biomedical and Health Sciences, Hiroshima University, 1-2-3 Kasumi, Minami-ku, Hiroshima, 734-8553 Japan; 4 Max Planck Institute for Psycholinguistics, Nijmegen, The Netherlands; 5 Oral and Maxillofacial Surgery II, Graduate School of Dentistry, Osaka University, Suita, Japan; 6 Section of Oral and Maxillofacial Radiology, UCLA School of Dentistry, Los Angeles, CA USA; 7 Department of Oral Pathology, Osaka University Graduate School of Dentistry, Suita, Osaka Japan; 8 3D Craniofacial Image Research Laboratory, University of Copenhagen, Copenhagen, Denmark

**Keywords:** Radiology, FMRI, Lymph node metastasis, Gingival cancer, Tumor signal intensity

## Abstract

**Objectives:**

This study sought to identify tumor characteristics that associate with regional lymph node metastases in squamous cell carcinomas originating in the upper gingiva.

**Materials and methods:**

Data from 113 patients from Osaka University Dental Hospital were included. We measured each primary tumor’s width, length, depth, and the extent of bone invasion. Additionally, tumor signal intensity for T1 and T2-weighted images as well as the center of the tumor’s location and T classification was assessed, and a histopathological analysis was performed.

**Results:**

Tumor signal intensity was not found to be a significant prognostic factor. However, bucco-lingual width, histopathological classification as well as the tumor’s location were significantly different between metastatic and non-metastatic groups in both univariate and multivariate analysis. Superior–inferior depth and T classification were significant only in the univariate (and not the multivariate) analysis.

**Conclusions:**

Bucco-lingual width, histopathological grading as well as the tumor’s location are likely to be important predictors for the occurrence of LN metastasis in upper gingival carcinoma patients and should be considered when managing care for these patients.

## Introduction

Head and neck cancers (HNCs) represent a serious global health issue. It is estimated that in 2020 there were more than 1.4 million new cases of HNC, which include cancers that originate in the oral or nasal cavity, the nasopharynx, the oropharynx the hypopharynx, the larynx, or the thyroid gland. Classical risk factors to develop HNCs are the use of tobacco and alcohol as they contain carcinogenic substances. Recently, it has also been shown that viruses such as the human papillomavirus (HPV) play an entirely different causal role compared to classical triggers (e.g., tobacco/alcohol) in the occurrence of HNCs, especially in the oropharynx [1].

More than 90% of cancers of the oral cavity and oropharynx are squamous cell carcinomas that arise in the squamous mucosal epithelium lining the mouth and throat [2]. These cancers most often arise in the tongue and floor of the mouth and are more prevalent in the mandibular mucosa (as compared to the maxillary mucosa) and occur less frequently in the gingiva (< 10%) [3].

A critical question for nearly all cancers, and in particular for squamous cell carcinomas (SCCs), is whether the cancer has metastasized to a distant site. The presence of metastases impacts prognosis and decreases the likelihood of successful curative therapy, often leaving palliative treatment as the only remaining option. Notably, SCCs are known for their high rate of lymph node (LN) metastasis [4, 5], and the presence of LN metastasis markedly reduces the survival rate [6, 7]. Thus, identification of regional LN metastasis in the neck is an important diagnostic evaluation in management of oral SCC.

Interestingly, gingival carcinomas have exhibited a relatively lower rate of LN metastasis [8] and published incidences range from 7 to 32% [9–11]. This is in marked contrast to other sites within the oral cavity (such as the tongue or the floor of the mouth) where the incidence of LN metastases is as high as 50% [12]. When LN metastasis is detected, surgical exploration and neck dissection is typically performed. For gingival carcinoma patients who have no signs of LN metastasis either no preventive treatment or an elective (precautionary) neck dissection is performed.

The primary tumor size may influence the probability of metastasis. Typically, tumor size is assessed clinically and with three-dimensional imaging methods, such as computed tomography (CT) and magnetic resonance imaging (MRI) [13, 14]. The most optimal method to detect a tumor’s bone invasion is CT [15, 16] whereas MRI is usually better for delineating tumor invasion into soft tissue [17]. Data from other tumor types including breast cancer, [18, 19] lung cancer [20], and endometrial cancer [21] shows that larger tumor sizes are associated with a higher likelihood of LN metastasis.

A recent meta-analysis of oral cancers showed that there is a relationship between cervical LN involvements and tumor thickness, with tumors over 4 mm being at significantly more at risk for metastasis [22]. However, the data in this meta-analysis was predominantly based on cancers originating in the tongue, buccal mucosa, and the lip, and did not encompass data from gingival SCC. Additionally, although there are reports of similar significant correlations between T-classification and the presence of regional metastasis from lower gingival SCC, [23, 24] few reports have investigated this issue for upper gingival cancers, which is the focus of the current study.

Clinicians typically use the “TNM-classification” (henceforth T-classification) [25]—a globally recognized standard, to categorize tumor size and spread. The T indicates the extent of the primary tumor, the N indicates involvement of lymph nodes, and the M indicates presence or absence of distant metastases. The T, N, and M components are further divided into categories (e.g. for “T” with a number indicating tumor extent, e.g. T1 < T2). In 2017 the classification system was significantly revised. Notably the parameter Depth of Invasion (DoI), as measured perpendicularly from the basement membrane to the deepest invasion point of the tumor, has been added to the oral cavity T-characterization to allow for the classification of cancers which have a small horizontal size but are nonetheless quite invasive into deeper tissues. With the new system, primary tumors which might have been earlier classified as T1 could now be scored as T2 if the DoI is more than 5 mm beyond the basement membrane. Likewise, a T2 score in the previous system could be upstaged to T3 if the DoI exceeds 10 mm.

The current study sought to identify which features can be associated with regional LN metastases in SCCs originating in the upper gingiva. In particular, investigating the behavior of SCCs in the upper gingiva is important—the anatomical positioning of the lymphatic system varies between the upper- and lower gingival areas [26], and thus upper gingival SCC may show a differential LN metastatic pattern than lower gingival SCC. To this end, we examined the tumor’s measured width, length, depth, its extent into bone, and imaging characteristics of the primary lesion, including MR signal intensity, image homogeneity on contrast-enhanced CT as these may provide additional useful insights into tumor behavior and its potential for LN metastasis.

## Materials and methods

### Patient selection

We analyzed data retrieved from our hospital’s radiology database from 2003 to 2017 and identified a total of 191 gingival cancer patients that were treated at Osaka University Dental Hospital. The inclusion criteria were: (1) patients that were clinically diagnosed with upper gingival cancer; (2) tumors were histopathologically confirmed by a pathologist to be SCCs; (3) CT and MRI scans were acquired no more than 10 days before treatment commenced; (4) written informed consent had to be obtained from all patients; and (5) patients had more than 2 years of follow-up. The exclusion criteria were: (1) no preoperative images were available; (2) artifacts interfered with image interpretation; (3) maxillary sinus cancer was present; and (4) patient had a previous history of cancer treatment. When applying all the above criteria, a total of 113 patients were ultimately included in this study. The current study abides by the Helsinki declaration and was approved by the ethics committee at Osaka University Graduate School of Dentistry (registered as study H21-E16). No conflict of interest was declared.

### CT and MR imaging

CT images were obtained using a 64-row multidetector CT scanner (Light Speed VCT; GE Healthcare, Milwaukee, WI). Images were taken at an axial plane parallel to the occlusal plane. Images were obtained at 120–140 kVp and 140–250 mA with a field of view of 25 × 25 cm and a matrix size of 512 × 512. The slice thickness was 0.625 mm without inter-slice gapping. An intravenous infusion of 100 mL Iohexol (Omnipaque 300, Daiichi-Sankyo Co., Tokyo, Japan), Iopamidol, (Iopamiron 300, Bayel Healthcare, Osaka, Japan) or Iomeprol (Iomeron 300, Eisai Co., Tokyo, Japan) was administered through an automated power injector. The injection sequence consisted of 70 mL at 0.6 mL/s, followed by an injection of 30 mL at 0.3 mL/s and simultaneous scanning.

MR images were acquired using a 1.5 T MR imaging scanner (Signa HDxt 1.5 T; GE Healthcare, Milwaukee, WI) equipped with an 8-channel phased array head and neck coil. The imaging protocol consisted of axial T1-weighted images (TR(msec)/TE(msec)/NEX; 500/7/1), axial T2-weighted images (3600/76/1), coronal T1-weighted images (500/7/1), coronal T2-weighted images (3600/76/1) using chemical shift selective (CHESS) fat suppression and contrast-enhanced axial and coronal or sagittal T1-weighted images with fat suppression (500/7/1) using the following parameters: 24 × 24 cm FOV; 256 × 256 matrix size; 5 mm section thickness; and 1 mm gap. The contrast media “gadopentetate dimeglumine” (Magnevist, Bayer Yakuhin Ltd.), gadodiamide (Omniscan, Daiichi Sankyo Co., Ltd.) or gadoteridol (Prohance, Eisai Co., Ltd.) were intravenously administered and consisted of an intravenous bolus injection at approximately 0.2 mL/kg body weight, followed by a 1 min delay and subsequent scanning.

### Image analysis

#### Measurement of the tumor size on MR images

**Fig. 1:**
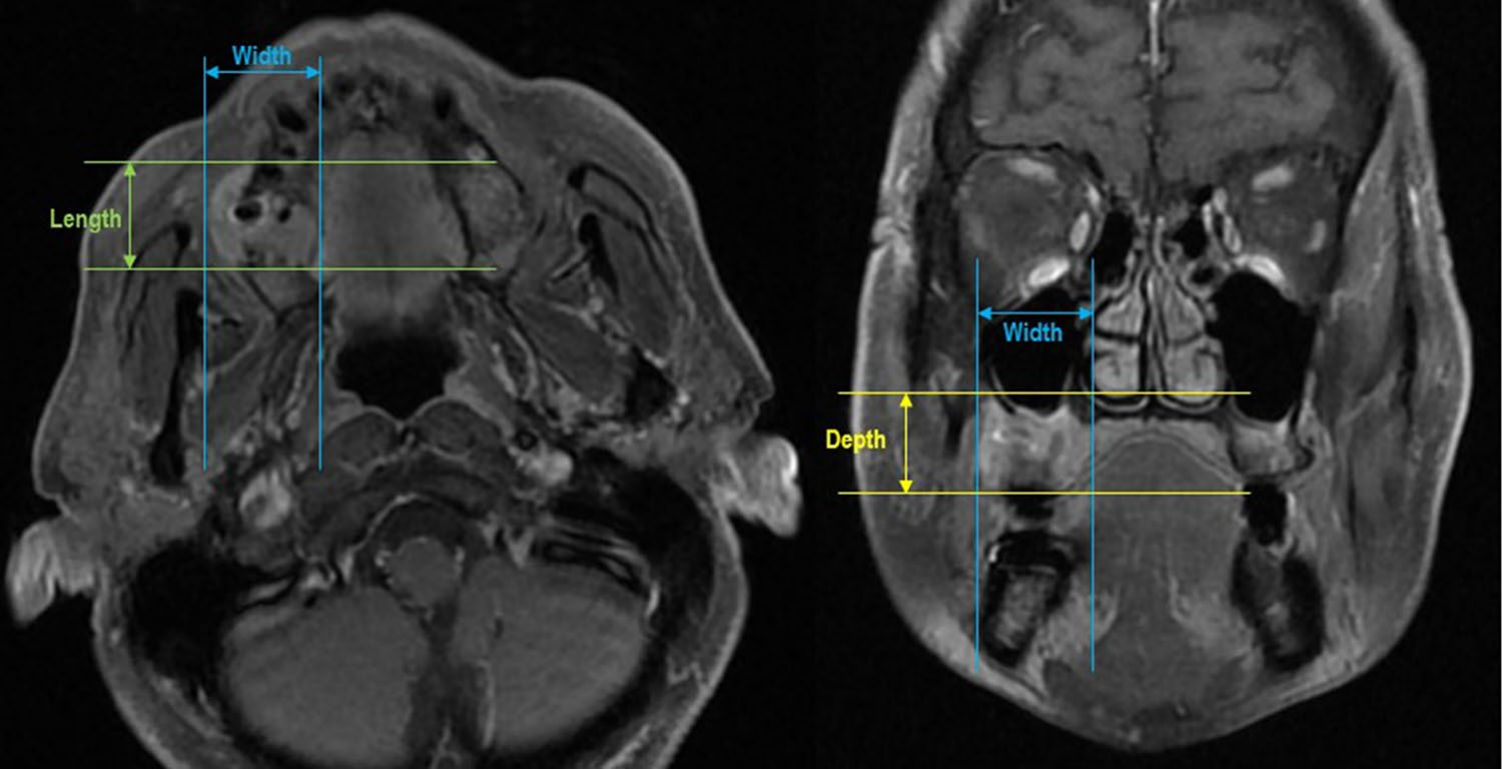
Orthogonal measurement of tumor size—non-metastatic patient. Tumor length and width were measured on axial MRI (left). Tumor depth and width were measured on coronal MRI (right). Both images were taken in a same patient where LN metastasis was NOT found

**Fig. 2:**
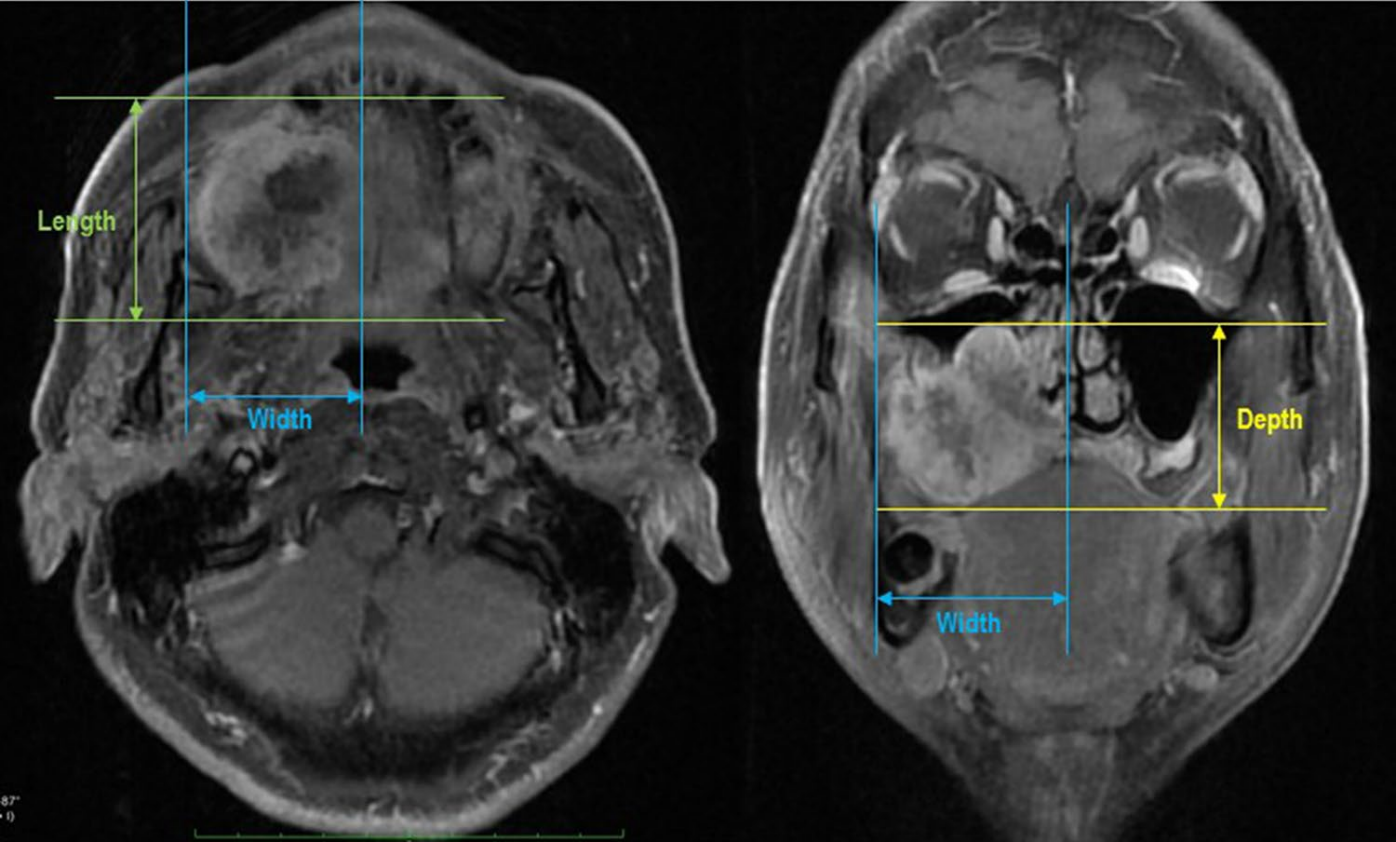
Orthogonal measurement of tumor size—metastatic patient. Tumor length and width were measured on axial MRI (left). Tumor depth and width were measured on coronal MRI (right). Both images were taken in a same patient where LN metastasis WAS found

Image evaluation was done by three experienced researchers. If an evaluation was not initially harmonious between the researchers, then all three researchers discussed it together to obtain clear consent. Tumor size was measured on T1 post-contrast images (see Figs. 1 and 2). Tumors were measured in the slice which showed the greatest tumor thickness in three orthogonal planes. Specifically, the bucco-lingual width and antero-posterior length were measured on axial and sagittal sections, and the superior-inferior depth was measured on coronal and sagittal sections.

### Tumor signal intensity on MR images

The tumor signal intensity was evaluated in comparison to the masseter muscle and parotid gland signal intensity on T1- and T2-weighted images to determine whether the tumor had a very high (super intense), slightly higher (hyperintense), equally intense (isointense) or lower (hypointense) signal intensity.

### Bone invasion

**Fig. 3:**
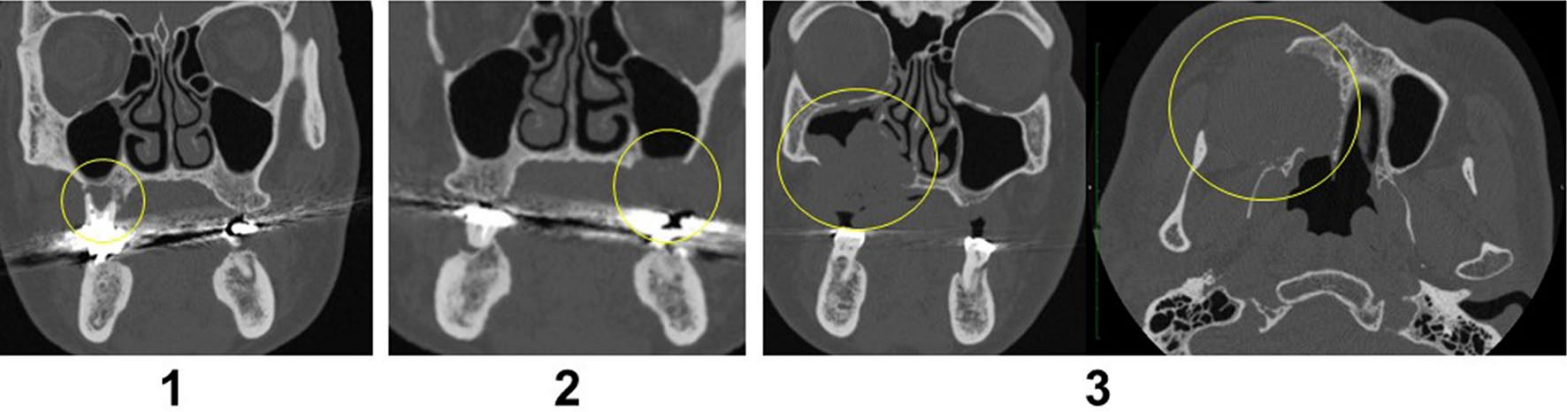
Bone Invasion Criteria. 0: no bone invasion, 1: invasion into maxilla, 2: invasion into sinus, 3: invasion into posterior maxillary wall. Bone invasion was diagnosed on axial, coronal, and sagittal CT images

Bone invasion was scored on CT images and categorized into one of the following four groups: (0) no invasion, (1) invasion into the alveolar process of the maxilla, (2) invasion into the sinus or nasal cavity, and (3) invasion into the posterior or lateral wall of the sinus or nasal cavity. See Fig. 3 for an overview of the criteria used.

### Histopathological lymph node (LN) metastasis

The final decision regarding LN status was made as follows: a LN was considered metastatic when histopathological examination during neck dissection confirmed the presence of metastasis. A LN was considered non-metastatic when it was found to be histopathologically clear of metastasis during neck dissection or when signs of LN metastasis were absent during more than 2 years of follow-up examinations (note: if signs of LN metastasis were present in follow-up examinations, they would be marked as metastatic).

### Statistical analysis

The patients were divided into two groups: LN-metastasis and no LN-metastasis. Differences in continuous variables between each of two groups were evaluated by the Mann–Whitney–Wilcoxon test. The relationship between, and independence among discrete variables of the ordinal scale between two and more groups in the univariate analysis were evaluated by the Chi-Square Test or Wilcoxon test. Multivariate logistic regression analysis was used to determine the multivariate correlation of LN metastasis. Significance levels were set at the 5% level. All analyses were carried out using R (R Core Team, 2019; http://www.R-project.org/; package: stats version 3.5.0) [27].

## Results

A total of 113 patients were included in this study; 56 were female and 57 were male. The mean age was 71.3 years (range 37–93). Neck dissection was performed at the initial stage in 52 of the 113 patients and out of these 52 patients, 25 had LN metastasis and 27 were found to be free of metastasis (which was histopathologically confirmed for each case). Sixty-one patients out of all included patients followed a “watch and wait” protocol and 10 out of those 61 patients developed occult (hidden) metastasis later. The total number of patients who acquired LN metastasis in this study was therefore thirty-five.

### Image analysis

#### Measurement of tumor size on images

Patients with LN metastasis had an average bucco-lingual tumor width of 28.8 ± 17.4 mm, an anterior–posterior length of 30.4 ± 15.1 mm, and a supero-inferior depth of 21.2 ± 13.0 mm. Patients without LN metastasis had an average bucco-lingual tumor width of 17.0 ± 9.7 mm, an anterior–posterior length of 26.3 ± 13.4 mm, and a supero-inferior depth of 16.6 ± 12.2 mm.

The bucco-lingual width (*W* = 821, *p* < 0.001) and the superior-inferior depth (*W* = 1015, *p* < 0.05) of the primary tumor of patients with LN metastasis were significantly greater than those of tumors without LN metastasis. However, there was no effect of the antero-posterior length (*W* = 1161, *p* = 0.21) between the two groups.

### Tumor signal intensity on MR images

From the total data set (*N* = 113), data from eighteen T1w MR images and nineteen T2w MR images signal intensity comparisons were not obtained due to technical concerns and/or insufficient image quality. Four evaluations of the relative tumor signal intensity were made (i.e., T1w vs. masseter muscle, T1w vs. parotid gland, T2w vs. masseter muscle, T2w vs. parotid gland). However, except for the T2w vs. parotid gland comparison, none of the other comparisons portrayed sufficiently large variability warranting subsequent analysis. Specifically, on T1w MR images, six tumors (6%) were super-intense, and 89 tumors (94%) were hyperintense when compared with the signal intensity of the masseter muscle. On T1w MR images, 100% of the tumors were isointense with the parotid gland. On T2w MR images, three tumors (3%) were hyperintense and ninety-two tumors (97%) were super-intense when compared with the masseter muscle signal intensity. Lastly, for T2w MR images, 15 tumors (16%) were isointense, 38 tumors (40%) were hypointense, and 41 tumors (44%) were super-intense when compared with the parotid gland signal intensity.

The relative signal intensities of the tumors did not differ between the metastatic and non-metastatic groups, except for the tumor signal intensity on T2w images, relative to the parotid gland. Although more tumors in the non-metastatic group generally yielded higher signal intensity compared with the metastatic group (16.7 vs. 5.7%), this was not statistically significant, *χ*_2_(3) = 2.7, *p* = 0.44. Consequently, signal intensity factors were deemed not to be predictive of metastasis and therefore not included in subsequent analyses.

### Univariate analyses

Tables 1 and 3 (third column) show an overview of the univariate results. With regards to tumor location, tumors in the metastatic group had centers located towards the molar region. More tumors in the metastatic group were poorly differentiated (42.9 vs. 12.8%), and with T4 stage (40 vs. 9%), both features that are commonly associated with a poorer prognosis. Lastly, bone invasion appeared to be more extensive for the metastatic group (Table 1), however, this difference was not statistically significant (*χ* = 6.10, *p* = 0.11).

**Table 1:** Descriptive information of the sample (split by metastatic- or non-metastatic group)

	Non-metastatic group	*N* = 78			
	Anterior	Premolar	Molar		
Center Location	17.9%	33.3%	48.7%		
	Well	Moderate	Poorly		
Histopathological analysis (differentiation)	46.2%	41.0%	12.8%		
	T1	T2	T3	T4a	T4b
T-classification	29.5%	34.6%	26.9%	9.0%	0%
	No-invasion	Maxilla	Sinus	Maxillary wall	
Bone invasion	26.9%	44.9%	19.2%	9.0%	
Age	71.4 ± 10.9				
Gender	Female: 40, male = 38				

	Metastatic group	*N* = 35			
	Anterior	Premolar	Molar		
Center location	5.7%	17.1%	77.1%		
	Well	Moderate	Poorly		
Histopathological analysis (differentiation)	22.9%	34.3%	42.9%		
	T1	T2	T3	T4a	T4b
T-classification	22.9%	25.7%	11.4%	22.9%	17.1%
	No-invasion	Maxilla	Sinus	Maxillary wall	
Bone invasion	17.1%	37.1%	20.0%	25.7%	
Age	71.2 ± 10.5				
Gender	Female: 16, male: 19				

### Multivariate analysis

Next, a multivariate logistic regression analysis was employed to determine which variables were significant in predicting metastasis. Unlike univariate analyses this type of analysis considers more than one predictive factor simultaneously which may show additional bearing to the variability of the dichotomous outcome (i.e., metastasis or no metastasis). The following variables were entered into the regression formula (see Table 2).

**Table 2:** Variables entered into the regression formula

Variables entered into the regression formula	Values	Details
Location of the center of the tumor	A: anterior region (i.e. incisor region including canine), P: premolar region, M: molar region	Metastasis from the primary lesion to regional lymph nodes might be related to the anatomical distribution of the lymphatic canals
Histopathological findings	1: Well differentiated, 2: moderately differentiated, 3: poorly differentiated	A measure which indicates how much cancer cells resemble the tissue they originated from
Bucco-lingual width	Actual measurement in mm	
Anterior–posterior length	Actual measurement in mm	
Superior-inferior depth	Actual measurement in mm	
T-classification	1: T1, 2: T2, 3: T3, 4: T4a, 5: T4b	TNM staging system as put forward by the Union for International Cancer Control (UICC)
Bone invasion	0: No invasion, 1: invasion into the maxilla, 2: invasion into the sinus, 3: invasion into the maxillary wall	Tumors having invaded into the sinus and the maxillary wall (which represent advanced cases)
Sex	Male/female	
Age	In years	

The multivariate logistic regression found that the location of the center of the tumor (estimated coefficient: 0.97, SE = 0.42, *z* = 2.31, *p* < 0.05), the histopathological findings (estimated coefficient: 1.01, SE = 0.36, *z* = 2.82, *p* < 0.01), and the bucco-lingual width of the tumor (estimated coefficient: 0.10, SE = 0.03, *z* = 2.90, *p* < 0.01) were predictive of the presence of LN metastasis. There were no interactions between length parameters (e.g., bucco-lingual width, anterior–posterior length, and superior-inferior depth), although the anterior–posterior length variable approached significance (estimated coefficient: − 0.07, SE = 0.04, *z* = − 1.80, *p* = 0.07) indicating a trend towards an incidence for LN metastasis. Importantly, superior-inferior depth as well as tumor class which were significant in the univariate analyses did not retain their significance in the multivariate analysis (indicating no additional predictivity over other significant factors entered in the analysis) (see Table 3).

**Table 3:** Results of the univariate and multivariate analysis

Variables entered into the regression formula	Values	Univariate analysis	Multivariate analysis
Location of the center of the tumor	A: anterior region (i.e. incisor region including canine), P: premolar region, M: molar region	*χ* = 8.18, *p* < 0.05	Est: 0.97, SE = 0.42, *z* = 2.31, *p* < 0.05
Histopathological findings	1: Well differentiated, 2: moderately differentiated, 3: poorly differentiated	*χ* = 13.05, *p* < 0.01	Est: 1.01, SE = 0.36, *z* = 2.82, *p* < 0.01
Bucco-lingual width	Actual measurement in mm	*W* = 821, *p* < 0.001	Est: 0.10, SE = 0.03, *z* = 2.93, *p* < 0.01
Anterior–posterior length	Actual measurement in mm	*W* = 1161, *p* = 0.21	Est: − 0.07, SE = 0.04, *z* = − 1.86, *p* = .06
Superior-inferior depth	Actual measurement in mm	*W* = 1015, *p* <0.05	Est: − 0.01, SE = 0.04, *z* = − 0.31, *p* = 0.75
T-classification	1: T1, 2: T2, 3: T3, 4: T4a, 5: T4b	*χ* = 20.49, *p* < 0.01	Est: 0.04, SE = 0.53, *z* = 0.08, *p* = 0.93
Bone invasion	0: No invasion, 1: invasion into the maxilla, 2: invasion into the sinus, 3: invasion into the maxillary wall	*χ* = 6.10, *p* = 0.11	Est: 0.48, SE = 0.60, *z* = 0.81, *p* = 0.42
Sex	Male/female	*χ* = 0.12, *p* = 0.73	Est: − 0.23, SE = 0.56, *z* = − 0.40, *p* = 0.69
Age	In years	*W* = 1377, *p* = 0.95	Est: − 0.004, SE = 0.03, *z* = − 0.15, *p* = 0.88

## Discussion

The current study aimed to identify tumor characteristics that could be related to regional lymph node metastases in SCCs which originated in the upper gingiva. We found several indicators which may predict LN metastases, including tumor center location, histopathological classification, bucco-lingual width, and a trend for anterior–posterior length.

The tumor center location showed a differential pattern for the metastasis patients vs. the non-metastasis patients. This fits well with the pattern found by Zhang et al. [28] who reported that tumor invasion into the gingivo-buccal sulcus was a significant risk factor for LN metastasis. They theorized that the rich lymphatic network in the buccal tissues might increase the likelihood of LN metastasis if gingival carcinoma would invade into this region.

Analysis of the histopathological classification identified noteworthy differences between the metastatic patients and the non-metastatic groups. Tumor cell differentiation is an indication of its biological behavior—well differentiated oral SCC cells resemble native gingival keratinocytes, and typically grow slower, relative to poorly differentiated tumor cells. Thus, our results show that histopathological cell differentiation is likely involved in the higher incidence of LN metastasis in upper gingival SCC patients. This result agrees with numerous studies of cancers at other sites, for example: skin cancer [29], squamous cell carcinoma of the oral cavity [30], gastric signet ring cell carcinoma [31], breast cancer [32] and lung cancer [33].

In several studies the T-classification is used as a measure of the size or extent of the primary tumor. However, in the current study this classification was not a significant prognostic factor for whether patients developed LN metastasis or not. This is likely resulting from the fact that in the metastasis group the patients were rather evenly spread amongst tumor class. For the non-metastasis group, though it was clear that the most advanced stages were less frequently present there was no overall statistically significant difference between the two groups.

We found that a greater bucco-lingual width of the primary tumor on MRI images was a significant predictor of LN metastasis, with a similar trend for increased anterior–posterior length. This might be since a larger bucco-lingual invasion diameter is indicative of a more aggressive tumor [22]. Additionally, Pentenero et al. [34] stated that it might be more difficult for tumor cells to enter the lymphatic system in superficial areas compared to the deeper soft tissue areas due the diameter difference between the areas (i.e., a wider diameter in the deeper areas might allow for easier access). This corroborates well with findings reported by Melchers et al. [35] who measured tumor depth in histological sections on 212 oral carcinomas (including 15 gingival carcinomas) and reported that it could indeed be a significant predictor of LN metastasis.

This study did not find any associations between tumor signal intensity on MR images and LN metastasis in this study (particularly the T2 versus Parotid Gland). Though signal intensity has been proposed to differentiate malignancies from other diseases [36], in our study no predictive relationship was found between tumor signal intensity and LN metastasis.

Lastly, there was no effect of Age or Sex between the two groups (as these were closely matched between groups) and we found no relationship between bone invasion in accordance with the American Joint Committee on Cancer (AJCC; i.e., particularly into the maxillary sinus and maxillary wall) and LN metastasis. One potential reason to explore whether bone invasion would be indicative of LN metastasis was because severe bone invasion would influence the T-classification.

## Conclusion

The bucco-lingual width, degree of tumor cell differentiation and the tumor’s location were important predictors of LN metastasis from upper gingival carcinoma. These parameters should be considered when evaluating imaging of SCC patients to enhance prognostic value and may lead to an improved treatment regimen.
