# Correction to: Prognostic factors for lymph node metastasis from upper gingival carcinomas

**DOI:** 10.1007/s11282-021-00575-x

**Published:** 2021-11-06

**Authors:** Mazen Aldosimani, Rinus G. Verdonschot, Yuri Iwamoto, Mitsuhiro Nakazawa, Sanjay M. Mallya, Naoya Kakimoto, Satoru Toyosawa, Sven Kreiborg, Shumei Murakami

**Affiliations:** 1 Department of Oral and Maxillofacial Radiology, Osaka University Graduate School of Dentistry, Suita, Osaka Japan; 2 Division of Radiology, Department of Oral Medicine and Diagnostic Sciences, College of Dentistry, King Saud University, Riyadh, Kingdom of Saudi Arabia; 3 Department of Oral and Maxillofacial Radiology, Institute of Biomedical and Health Sciences, Hiroshima University, 1-2-3 Kasumi, Minami-ku, Hiroshima, 734-8553 Japan; 4 Max Planck Institute for Psycholinguistics, Nijmegen, The Netherlands; 5 Oral and Maxillofacial Surgery II, Graduate School of Dentistry, Osaka University, Suita, Japan; 6 Section of Oral and Maxillofacial Radiology, UCLA School of Dentistry, Los Angeles, CA USA; 7 Department of Oral Pathology, Osaka University Graduate School of Dentistry, Suita, Osaka Japan; 8 3D Craniofacial Image Research Laboratory, University of Copenhagen, Copenhagen, Denmark

## Correction to: Oral Radiology 10.1007/s11282-021-00568-w

The article Prognostic factors for lymph node metastasis from upper gingival carcinomas, written by Mazen Aldosimani, Rinus G. Verdonschot, Yuri Iwamoto, Mitsuhiro Nakazawa, Sanjay M. Mallya, Naoya Kakimoto, Satoru Toyosawa, Sven Kreiborg and Shumei Murakami, was originally published electronically on the publisher’s internet portal on 24 September 2021without open access. With the author(s)’ decision to opt for Open Choice the copyright of the article changed on 21-October-2021 to © The Author(s) 2021 and the article is forthwith distributed under a Creative Commons Attribution 4.0 International License, which permits use, sharing, adaptation, distribution and reproduction in any medium or format, as long as you give appropriate credit to the original author(s) and the source, provide a link to the Creative Commons licence, and indicate if changes were made. The images or other third party material in this article are included in the article’s Creative Commons licence, unless indicated otherwise in a credit line to the material. If material is not included in the article’s Creative Commons licence and your intended use is not permitted by statutory regulation or exceeds the permitted use, you will need to obtain permission directly from the copyright holder. To view a copy of this licence, visit http://creativecommons.org/licenses/by/4.0.

The original article has been corrected.

